# WEE1 and PARP-1 play critical roles in myelodysplastic syndrome and acute myeloid leukemia treatment

**DOI:** 10.1186/s12935-023-02961-3

**Published:** 2023-06-27

**Authors:** Seiichi Okabe, Yuko Tanaka, Mitsuru Moriyama, Akihiko Gotoh

**Affiliations:** Department of Hematology, Tokyo Medical University, 6-7-1 Nishi-shinjuku, Shinjuku-ku, 160-0023 Tokyo, Japan

**Keywords:** Cell proliferation, Myelodysplastic syndrome, PARP-1, WEE1

## Abstract

**Background:**

Myelodysplastic syndrome (MDS) is a clonal bone marrow disorder defined by cytopenia and is associated with an increased risk of transformation to acute myeloid leukemia (AML). The outcome of MDS is poor, so alternative therapeutic approaches are needed to improve survival. The inhibition of the DNA damage response pathway, including poly (ADP-ribose) polymerase-1 (PARP-1), has been approved to treat several cancers. In addition, WEE1, a nuclear kinase, is overexpressed in many cancers. Therefore, a WEE1 inhibitor combined with a PARP-1 inhibitor could inhibit the proliferation of MDS and AML.

**Methods:**

We analyzed whether WEE1 was regulated in the progression of MDS and AML. We also evaluated the efficacy of MK-1775 (WEE1 inhibitor) and talazoparib (PARP-1 inhibitor).

**Results:**

PARP-1 expression was higher in the AML cells than in the MDS cells. However, WEE1 expression remained unchanged. MK-1775 or talazoparib alone inhibited MDS and AML cells after 72 h, and cellular cytotoxicity and caspase 3/7 activity were increased. The combined use of MK-1775 and talazoparib produced superior efficacy than either drug alone and SKM-1 colony formation was reduced. Significant cell populations in the sub-G1 phase were found in the cell-cycle analyses. Additionally, γ-H2AX expression and caspase 3 activity were increased. The combined treatment also changed the mitochondrial membrane potential.

**Conclusions:**

The combination of a WEE1 inhibitor and PARP-1 inhibitor had enhanced efficacy and is proposed as a new therapeutic option for patients with MDS or AML. Our findings have clinical implications for a potential novel therapeutic strategy for MDS and AML patients.

**Supplementary Information:**

The online version contains supplementary material available at 10.1186/s12935-023-02961-3.

## Background

Myelodysplastic syndrome (MDS) is a clonal bone marrow disorder defined by cytopenia and an increased risk of developing acute myeloid leukemia (AML) [1]. MDS is more common in the elderly [2]. MDS has various clinical manifestations and prognoses, and the international prognostic scoring system (IPSS) and its revised version (IPSS-R) are used in the clinical management of MDS patients [3]. The treatment for individuals with MDS is driven by disease risk, assessed according to the IPSS and IPSS-R. Hypomethylating agents (HMAs) have traditionally been the primary front-line therapy for high-risk patients with MDS [3]. Azacitidine was shown to significantly improve the overall survival of MDS patients in the AZA-100 trial [4]. However, none of the current MDS treatment options are curative, except for allogeneic hematopoietic cell transplantation [3]. High-risk MDS is associated with a major risk of progression to AML and short survival [5]. Although HMAs are used to treat high-risk MDS patients, patients who fail to respond to HMAs have a very poor survival (median < 6 months) [5]. Therefore, an alternative strategy is required to improve the prognosis of patients with MDS, especially high-risk patients.

Targeting DNA damage is considered an appropriate cancer-treatment strategy. WEE1, a serine-threonine kinase located in the cell nuclei, regulates the G2/M checkpoint [6]. WEE1 is activated by the DNA damage response (DDR) and triggers G2/M arrest. In preclinical models, WEE1 inhibitors have increased the sensitivity to chemotherapy or radiotherapy, particularly in p53-mutant or p53-deficient cancer cells [7]. A previous report demonstrated that cell-cycle checkpoint proteins, particularly WEE1, are critical mediators of AML cell survival after cytarabine exposure [8]. In AML cells, *WEE1* is a key gene discriminating between *FLT*-ITD, *FLT*-TKD, and *NRAS*-mutated samples. Moreover, a previous report demonstrated that the WEE1 inhibitor AZD1775 combined with HDAC inhibitors targeted human acute myeloid leukemia cells harboring various genetic mutations, including p53-wild type, p53-deficient, and FLT3-ITD leukemia cells [9]. Poly (ADP-ribose) polymerase (PARP) is involved in the DDR [10] and is a family of proteins required for several cellular processes, including programmed cell death. A previous report demonstrated that PARP inhibitor-induced lethality in leukemia was driven by AML1-ETO and PML-RARA. AML cells with low expressions of key members of the DDR pathway, such as Rad51, ATM, BRCA1, and BRCA2, displayed obvious sensitivity to PARP inhibitors [11]. Since PARP helps repair DNA when damaged, we hypothesize that PARP inhibitors may enhance WEE1 inhibition in MDS and AML cell lines.

In the present study, we investigated how MK-1775 (WEE1 inhibitor) affected MDS and AML cells. Additionally, we evaluated whether the co-treatment of MK-1775 and talazoparib (PARP inhibitor) increased the cytotoxicity in MDS and AML cell lines.

## Methods

### Reagents

The WEE1 inhibitor, MK-1775, was obtained from Selleck Chemicals (Houston, TX, USA). Talazoparib (BMN-673), an orally active PARP-1/2 inhibitor, was purchased from MedChemExpress (Monmouth Junction, NJ, USA). MK-1775 and talazoparib stock solutions were dissolved in dimethyl sulfoxide. All other reagents were obtained from Merck KgaA (Darmstadt, Germany).

### Cell lines

SKM-1 (MDS cell line), MOLM-14 and Kasumi-1 (AML cell lines), and NIH3T3 (Mouse fibroblast-like cell line) cells were purchased from the Japanese Collection of Research Bioresources Cell Bank (Ibaraki, Osaka, Japan). U937, THP-1, and MV4-11 (AML cell lines) cells and the human marrow stromal cell line HS-5 were purchased from the American Type Culture Collection (Manassas, VA, USA). MDSL (MDS cell line) cells were kindly provided to us by Professor Kaoru Tohyama (Kawasaki Medical School, Kurashiki City, Okayama, Japan). These cell lines were grown in Roswell Park Memorial Institute 1640 (RPMI 1640) medium, which contained 10% fetal bovine serum (FBS) and 1% penicillin/streptomycin. MDS-L cells were cultured in RPMI 1640 medium with 20% FBS. NIH3T3 and HS-5 cells were cultured in Dulbecco’s modified Eagle’s medium (DMEM) with 10% FBS.

### Cell proliferation assay

For the cell proliferation assay, 2 × 10^5^ cells/ml were treated with MK-1775 and/or talazoparib for 72 h. Cell viability was measured by a cell counting kit-8 (Dojindo Laboratories, Mashikimachi, Kumamoto, Japan) at 450 nm or a CellTiter-Glo® Luminescent Cell Viability Assay (Promega, Madison, WI, USA) using an EnSpire Multimode Plate Reader (PerkinElmer, Waltham, MA, USA) according to the manufacturer’s protocol.

### Caspase 3/7 activity

A Caspase Glo 3/7 assay kit (Promega, Madison, WI, USA) was used for measuring the caspase activity according to the manufacturer’s instructions. The luminescence of each sample was analyzed using an EnSpire Multimode Plate Reader after 48 h of incubation with the indicated concentrations of MK-1775 and/or talazoparib.

### Short-hairpin RNA (shRNA) transfection

The mammalian *WEE1* gene expression lentiviral vector and the control shRNA vector were obtained from VectorBuilder Japan, Inc. (Yokohama, Kagawa, Japan). SKM-1 cells were cultured in a six-well culture dish for 24 h in RPMI 1640 medium with 8 g/mL hexadimethrine bromide (Sigma-Aldrich) and were infected with the lentiviral vectors. The medium was replaced the next day with new complete media. Immunoblotting was performed to determine the expression of WEE1.

### Mitochondrial membrane potential

A mitochondria Staining Kit (Merck KgaA) was used to analyze the mitochondrial membrane potential according to the manufacturer’s protocol. After 72 h of incubation with MK-1775 and/or talazoparib, JC-1 monomers and aggregates were analyzed using a plate reader.

### Cytotoxicity assay

Cells were incubated for 72 h with the indicated concentrations of MK-1775 and/or talazoparib. A Cytotoxicity LDH Assay kit with water-soluble tetrazolium salt was used to assess the cytotoxic effects of MK-1775 and/or talazoparib on the leukemia cells based on LDH release (Dojindo Laboratories). An EnSpire Multimode Plate Reader was used to measure the amount of LDH released from the dead cells.

### Cell cycle analysis

The cell cycle was determined using a BD CycletestTM Plus DNA Reagent Kit (Becton-Dickinson, Mountain View, CA, USA) according to the manufacturer’s protocol. SKM-1 cells were cultured for 24 h in the presence of MK-1775 (100 nM) and/or talazoparib (1 µM). The distribution of the DNA content was examined using a BD FACSVerseTM Flow Cytometer (Becton-Dickinson) and analyzed with BD FACSuite software (Becton-Dickinson).

### Reactive oxygen species (ROS) assay

SKM-1 cells were cultured in RPMI medium with MK-1775 and/or talazoparib to investigate the ROS activity. After 24 h, the cells were harvested, and the ROS activity was analyzed using an ROS Assay Kit-Highly Sensitive DCFH-DA kit (Dojindo) according to the manufacturer’s protocol. An EnSpire Multimode Plate Reader was used to measure the ROS activity.

### Adenosine triphosphate (ATP) assay

A Cell ATP test reagent Ver.2 kit (TOYO B-Net, Tokyo, Japan) was used to measure the intracellular ATP following the manufacturer’s instructions. An EnSpire Multimode Plate Reader was used to calculate the ATP levels.

### Colony assay

Colony assays were carried out in accordance with the manufacturer’s instructions and described previously [12]. In brief, 1 × 10^2^ SKM-1 cells were plated in triplicate in six-well plates containing a methylcellulose medium (MethoCultTM Express # 04437; StemCell Technologies, Vancouver, Canada) and either MK-1775 or talazoparib. The plates were incubated in a 5% CO_2_ incubator at 37 °C. The colony counts were determined using an EVOSTM FL Digital Inverted Fluorescence Microscope seven days after plating (ThermoFisher Scientific, Inc., Waltham, MA, USA). The experiments were carried out three times, and the results are presented as the mean and standard error.

### Immunoblots

The immunoblot analyses were carried out using the previously described methods [13, 14]. Briefly, the cells were incubated with MK-1775 and/or talazoparib at the indicated concentrations for 24 h. The cells were washed with ice-cold phosphate-buffered saline (PBS) and lysed with radioimmunoprecipitation lysis buffer. The protein concentrations were measured using a Bio-Rad Protein Assay kit (Bio-Rad, Hercules, CA, USA). Samples were loaded on 4–20% mini protein TGX gels and separated by electrophoresis. The gels were then transferred to polyvinylidene difluoride membranes (Millipore, Billerica, MA, USA). The membranes were blocked and incubated for one hour with the primary antibodies at the appropriate dilutions. The blots were washed, incubated with the secondary antibodies, and developed with a chemiluminescence system (Amersham Pharmacia Biotech Ltd, Little Chalfont, UK). Anti-phospho-histone H2A.X (Ser139) (Merck KgaA), cleaved caspase 3 and cleaved PARP (Cell Signaling Technology, Danvers, MA, USA), and WEE1 and β-actin (Santa Cruz Biotechnology, Santa Cruz, CA, USA) were used as the primary antibodies. Three separate experiments were carried out.

### Statistical analyses

The prism 9 software (GraphPad Software, San Diego, CA, USA) was used to analyze all the presented data. Two-tailed Student’s *t*-tests were used for testing statistical significance. If one of the groups in the study was considered the control group, data were analyzed by Dunnett’s test as the post-hoc test following an ANOVA. When comparing three or more samples, data were analyzed by a one-way ANOVA with Turkey post hoc comparison tests with an alpha of 0.05 and an *n* of 3 or more. Significance was expressed as *p < 0.05, **p < 0.01, ***p < 0.001, ****p < 0.0001.

## Results

### Gene expression in MDS and AML cells

**Fig. 1:**
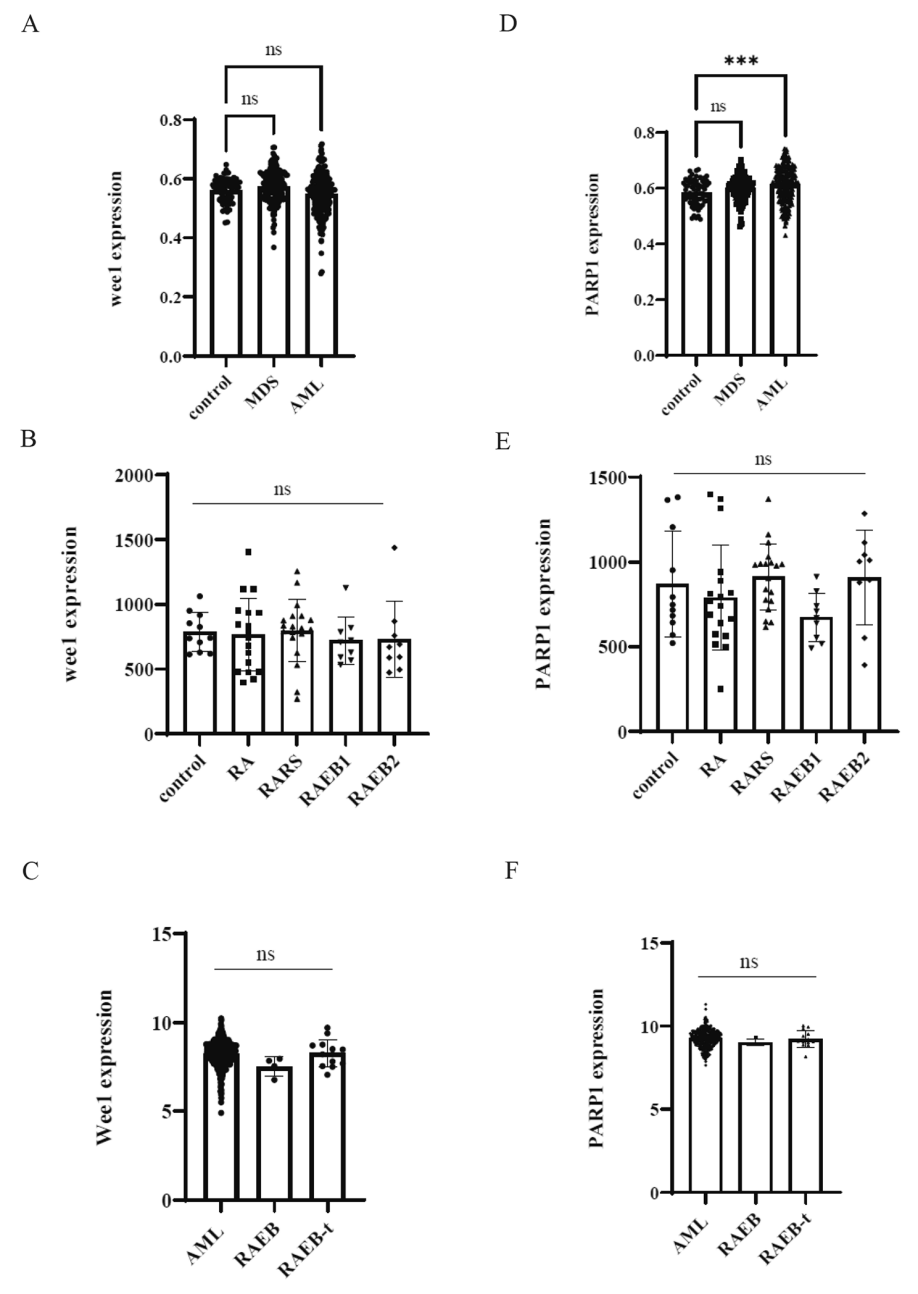
Gene expressions of *WEE1* and *PARP1* in the MDS or AML cell lines. (**A, D**) The gene expressions of *WEE1* and *PARP1*. The validation of the *WEE1* gene by the GEO data (GSE15061) comparing the 164 MDS, 202 AML, and 69 non-leukemia bone marrow samples (control group). (**B, E**) The gene expressions of *WEE1* and *PARP1* by the GEO data (GSE4619) comparing the MDS patient group (*n* = 55; 18 patients had RA, 19 had RARS, 9 had RAEB1, and 9 had RAEB2) and the control group (*n* = 11). (**C, E**) The gene expression data of the *WEE1* and *PARP1* genes by the GEO data (GSE12211) comparing the AML patient group (*n* = 443), RAEB group (*n* = 4), and the RAEB-t group (*n* = 13). Significance was expressed as ***p < 0.001 and ns: not significant

WEE1 is a key gatekeeper for the G2/M checkpoint and is involved in DNA damage repair [6]. The *WEE1* gene encodes a nuclear tyrosine kinase belonging to the Ser/Thr protein kinase family [9]. We hypothesized that the *WEE1* gene is involved in MDS and AML disease progression. We first investigated *WEE1* expression using a public functional genomics database. According to the Gene Expression Omnibus database (National Center for Biotechnology Information, Bethesda, MD, USA), *WEE1* gene expression was not increased in MDS and AML cells compared with the normal control sample (GSE15061) (Fig. 1A). From the GSE4619 data, *WEE1* gene expression was unchanged, according to the MDS staging (by the French American British classification), from refractory anemia to refractory anemia with excess blasts (RAEB) (Fig. 1B). *WEE1* gene expression seems to increase in AML cells compared with the expression in MDS RAEB samples; however, it was not statistically significant (GSE14468) (Fig. 1C).

The PARP enzyme is known to affect transcription and various DNA repair pathways [10]. Recently, PARP inhibitors have stood out among new drugs targeting DNA repair in conditions such as ovarian and breast cancers [15]. Thus, we investigated the gene expression of *PARP1* in the MDS and AML samples. The *PARP1* gene was increased in the AML samples compared with the normal samples (Fig. 1D). However, *PARP1* expression was unchanged in the MDS samples (Fig. 1E F).

### Cell viability of MDS and AML cell lines following MK-1775 or talazoparib exposure

Although *WEE1* gene expression was unchanged in the MDS and AML samples, *PARP1* gene expression was increased in the AML samples from the GEO data. Induction of replication stress combined with the selective abrogation of DNA damage repair and DNA damage checkpoints in cancer cells represents an anticancer strategy [16]. A previous report demonstrated that the combination of a WEE1 inhibitor and a PARP inhibitor-induced replication stress and DNA damage in ovarian cancer and KRAS mutated non-small cell lung cancer [16, 17]. A previous report also investigated the response to MK-1775 and talazoparib, a highly potent orally active PARP-1/2 inhibitor used to treat breast cancer [18]. Therefore, we first tested different concentrations of the WEE1 and PARP inhibitors by using MDS and AML cell lines, including SKM-1. Although SKM-1 cells were isolated and established from MDS patients a long time ago [19], this cell line was used in the public data portal resource for the acceleration of cancer research using model cancer cell lines [20].

**Fig. 2:**
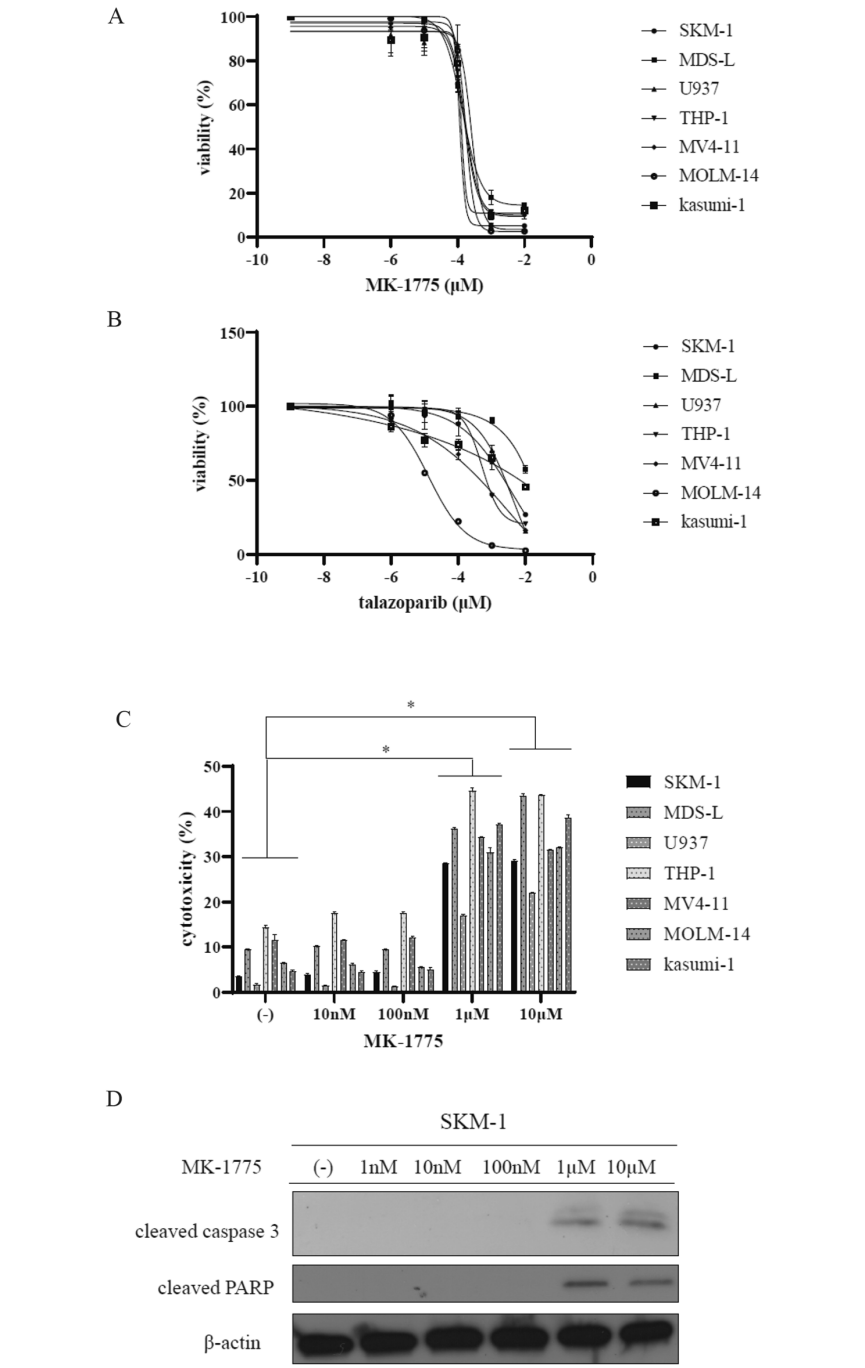
Effects of MK-1775 or talazoparib on the MDS and AML cell lines. (**A, B**) MDS and AML cell lines were cultured in RPMI 1640 medium supplemented with 10% FBS or 20% FBS, respectively, and with MK-1775 or talazoparib for 72 h. Cell growth was evaluated with a cell counting kit-8. (**C**) The MDS and AML cell lines were treated with MK-1775 for 72 h. The cytotoxicity was analyzed using a Cytotoxicity LDH Assay kit. ^*^p < 0.05 vs. the control. (**D**) SKM-1 cells were treated with MK-1775 for 24 h. The total extracts were examined by immunoblot analysis with antibodies against cleaved caspase 3, cleaved PARP, and β-actin

Our results showed that the WEE1 inhibitor, MK-1775 (Fig. 2A), and talazoparib (Fig. 2B) inhibited the proliferation of all the MDS and AML cell lines. However, the sensitivity of talazoparib was different between the cell lines (Fig. 2B). We next investigated the cytotoxicity using an LDH-based assay to determine the percentage of dead cells. MK-1775 increased the percentage of cytotoxicity in a dose-dependent manner (Fig. 2C). MK-1775 activated caspase 3 and PARP in the MDS cell line, SKM-1, in a dose-dependent manner, according to the immunoblot analysis (Fig. 2D).

### Co-treatment with the WEE1 and PARP-1 inhibitors impaired colony formation

**Fig. 3:**
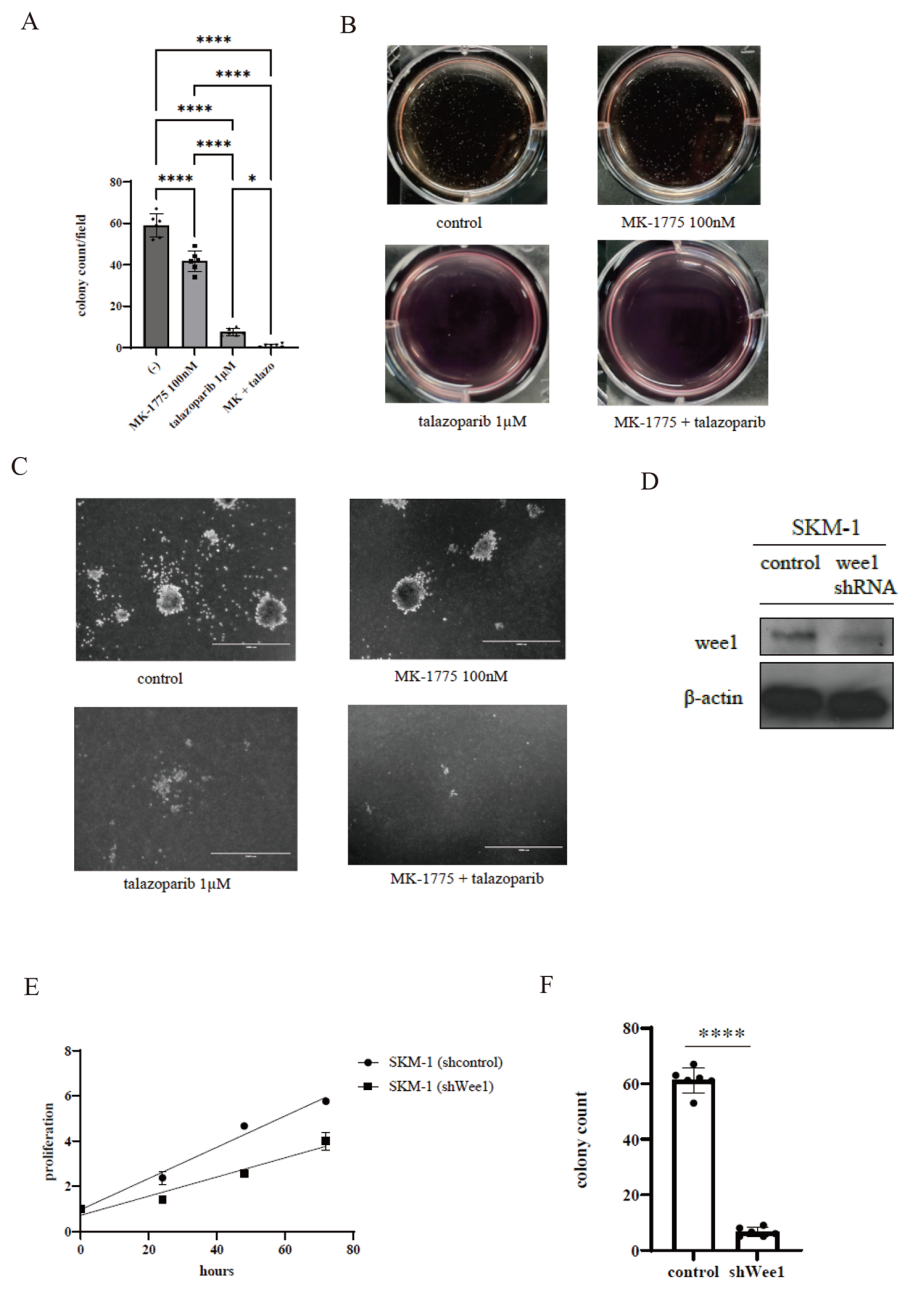
Colony formation assay of the SKM-1 cell line and the analysis of the cell proliferation by WEE1 shRNA transfection. (**A, B, C**) Cells were treated with 100 nM MK-1775 and/or 1 µM talazoparib for seven days. The colonies per dish were photographed using a digital camera and counted using an EVOS™ FL Digital Inverted Fluorescence Microscope. The quantification graph displaying the colony formation and representative images from three independent sets of experiments are shown. Scale bar: 1,000 μm. The results represent three independent experiments. (**D**) Total extracts of shRNA-transfected SKM-1 cells were examined by immunoblot analysis using antibodies against WEE1 and β-actin. (**E**) Cellular proliferation of shRNA-transfected SKM-1 cells was evaluated with a cell counting kit-8. (**F**) shRNA-transfected SKM-1 cells (1 × 10^2^ cells) were plated in triplicate on dishes containing a methylcellulose medium. The colony numbers were calculated. Significance was expressed as *p < 0.05, **p < 0.01, ***p < 0.001, and ****p < 0.0001

We found that the colony count was reduced by combining MK-1775 with talazoparib (Fig. 3A). The bright field image displays confirmed that SKM-1 colonies were reduced by the cotreatment of MK-1775 and talazoparib, compared with each drug alone (Fig. 3B, C). Moreover, the expression level of WEE1 in the SKM-1 cells was reduced compared with the controls (Fig. 3D). A final concentration of 2 × 10^5^ cells/ml was incubated, and the cell proliferation was compared during the exponential phase and after the lag phase. We found that cell proliferation was reduced in the shRNAtransfected SKM-1 cells compared with the control shRNA-transfected cells (Fig. 3E). The shRNA-transfected cells were possibly selected from the cells that escaped the apoptosis induced by the WEEl reduction and may have reduced the proliferation. The colony counts were also reduced (Fig. 3F). These results indicate that *WEE1* is involved in the cell proliferation of SKM-1 cells.

### MK-1775 and talazoparib inhibited the growth of the MDS cell lines

**Fig. 4:**
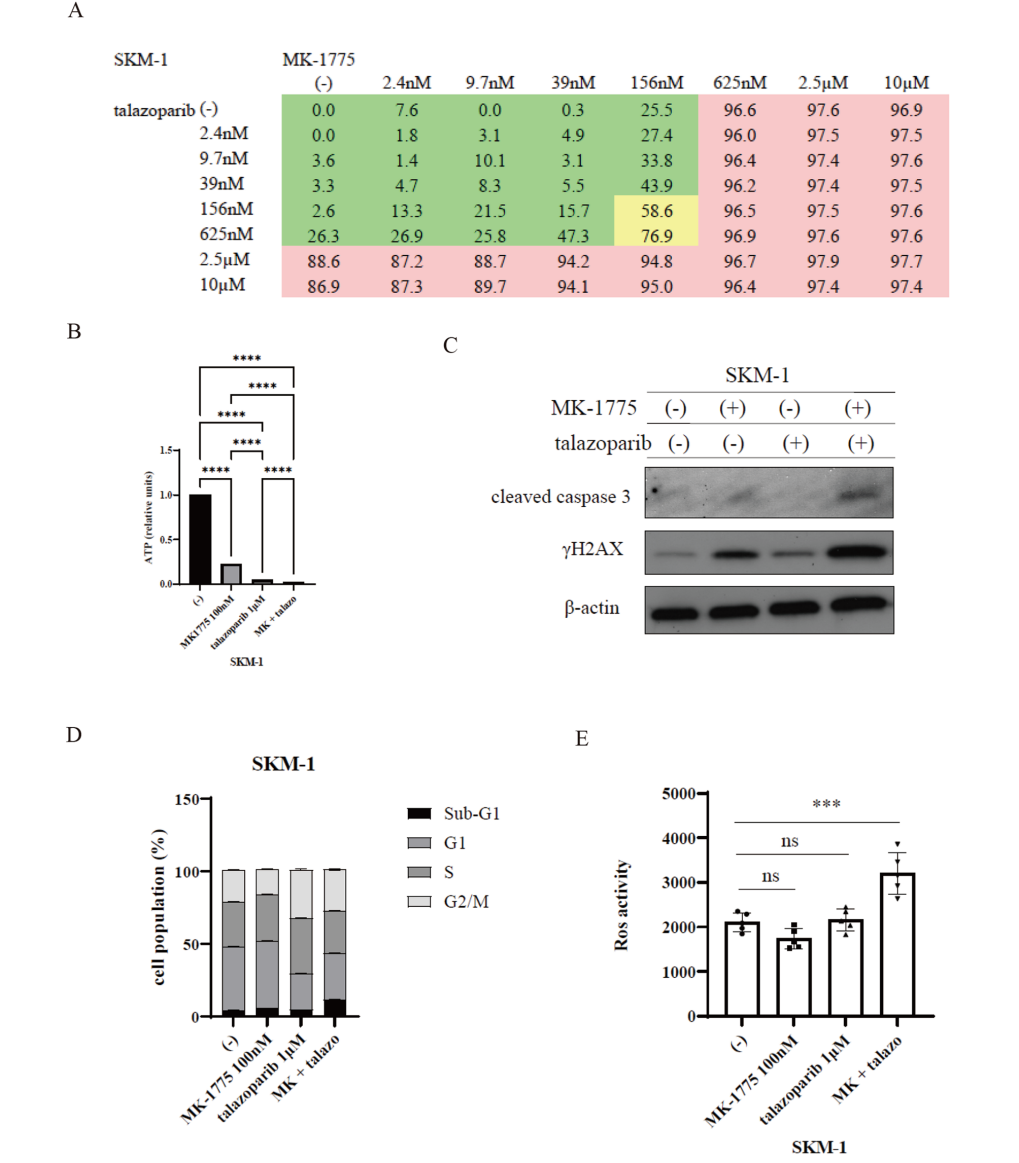
MK-1775 combined with talazoparib induced cytotoxicity in the MDS cells. SKM-1 cells were treated with MK-1775 and/or talazoparib for 72 h. Cellular proliferation was evaluated with a cell counting kit-8. (**A**) MDS cells were incubated with MK-1775 or talazoparib for 72 h. The intracellular ATP levels were determined using a Cell ATP assay reagent Ver.2 kit. (**B**) SKM-1 cells were treated with MK-1775 and/or talazoparib for 24 h. The total extracts were examined by immunoblot analysis with antibodies against γ-H2AX, cleaved caspase 3, and β-actin. (**C**) Cell cycle phase profiling was determined by a BD Cycletest™ Plus DNA Reagent Kit using SKM-1 cells treated with MK-1775 and/or talazoparib for 24 h. A representative histogram for each condition is illustrated. (**D**) SKM-1 cells were cultured in RPMI medium with MK-1775 and/or talazoparib for 24 h. ROS activity was analyzed using a ROS Assay Kit -Highly Sensitive DCFH-DA, according to the manufacturer’s protocol. Significance was expressed as *p < 0.05, **p < 0.01, ***p < 0.001, ****p < 0.0001, and ns, not significant

The Co-treatment of MK-1775 and talazoparib inhibited cell growth more than either drug alone. The combination index (CI) by Chou-Talalay provided quantitative information [21]. Since the CI values for MK-1775 plus talazoparib were less than 1.0, the drug combination was likely synergistic in this experiment (Fig. 4A). Since ATP is considered the molecular unit of intracellular energy [22] and is used as a controllable source of energy in cells, the levels of this compound offer a potential marker for cell viability and growth [23]. Therefore, we evaluated the intracellular ATP levels. The co-treatment of MK-1775 and talazoparib reduced the amount of ATP in the MDS cell line (Fig. 4B). The immunoblot analysis revealed that γ-H2AX expression was increased after the co-treatment of MK-1775 and talazoparib. Cleaved-caspase 3 and cleaved-PARP levels were also increased (Fig. 4C). SKM-1 cells incubated with MK-1775 for 24 h had an increase in the G1 phase compared with the controls. Additionally, the co-treatment of MK-1775 and talazoparib resulted in a higher sub-G1 population (Fig. 4D, Supplemental Fig. 1). Further, we found that ROS was increased after the co-treatment of MK-1775 and talazoparib compared with each drug alone (Fig. 4E).

### MK-1775 and talazoparib induced cell death in the MDS and AML cell lines

**Fig. 5:**
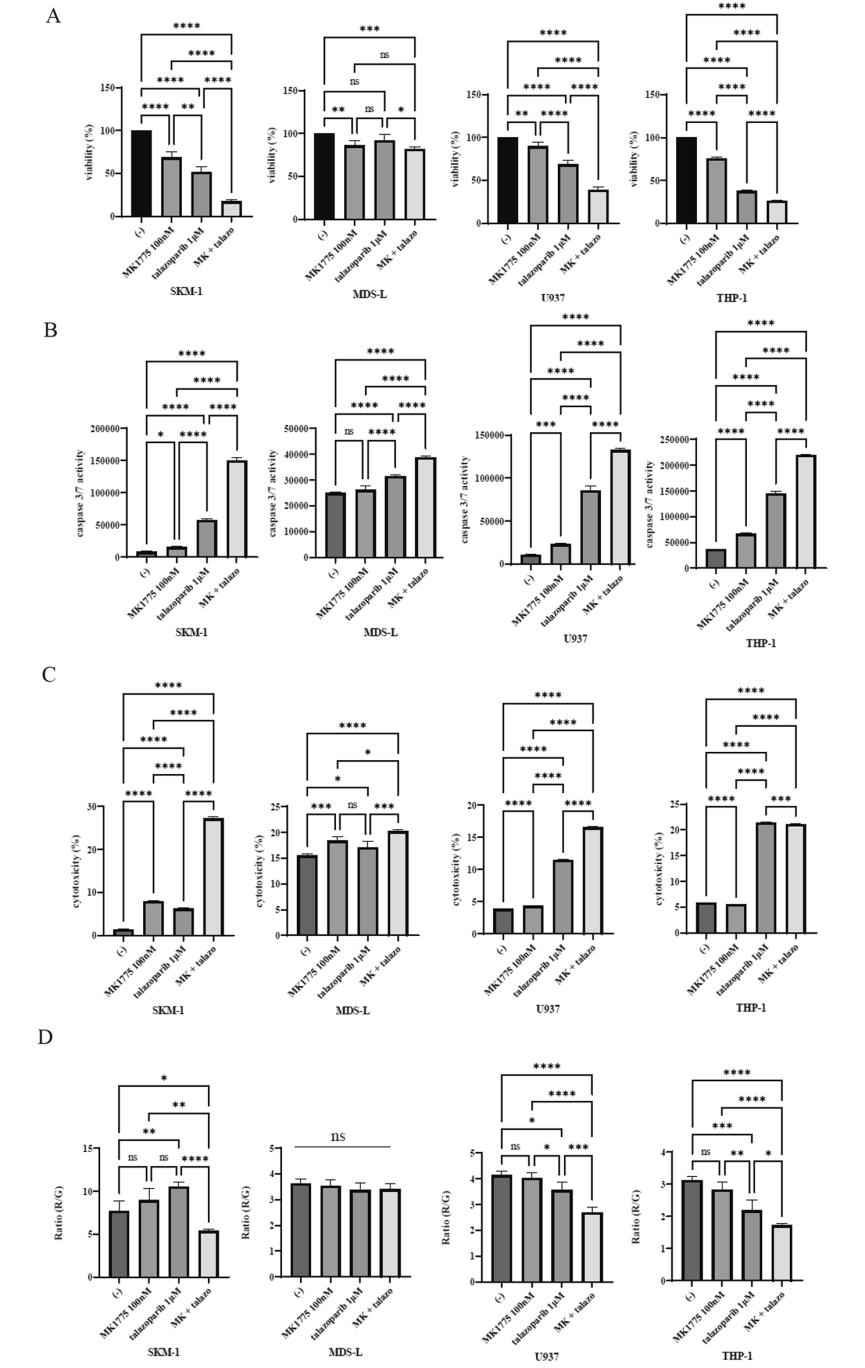
Effects of MK-1775 and talazoparib on MDS and AML cell proliferation. (**A, B, C**) MDS and AML cell lines were incubated with MK-1775 or talazoparib for 48 or 72 h. Cell growth (**A**), caspase 3/7 activity (**B**), and cytotoxicity (**C**) were evaluated. ^*^p < 0.05 vs. the control. (**D**) MDS and AML cell lines were incubated with MK-1775 or talazoparib for 72 h. The MMP was analyzed by a Mitochondria Staining Kit. Significance was expressed as *p < 0.05, **p < 0.01, ***p < 0.001, ****p < 0.0001, and ns, not significant

To evaluate the cellular response to WEE1 and PARP-1 inhibition, we used a cell viability assay. The cells were exposed to MK-1775 and/or talazoparib for 72 h. As shown in Fig. 5A and Supplemental Fig. 2A, the cell viability was reduced by the co-treatment of MK-1775 and talazoparib. In contrast, the cell viabilities of the non-cancer cell lines, NIH3T3, and HS-5, were unchanged after the co-treatment of MK-1775 and talazoparib (Supplemental Fig. 2A). We also found that the caspase 3/7 activity, a reliable indicator of apoptosis, was increased by combining talazoparib and MK-1775 (Fig. 5B, Supplemental Fig. 2B). In addition, the cytotoxicity was increased by combining talazoparib and MK-1775 (Fig. 5C, Supplemental Fig. 2C). The mitochondria membrane potential (MMP) is required for ATP production and is a key indicator of mitochondrial activity [24]. Mitochondrial function is a critical indicator of overall cell health, highlighted by the association of mitochondrial dysfunction and various diseases, including cancer [25]. Thus, we investigated the MMP by using a Mitochondria Staining Kit. We found that the MMP was decreased after the co-treatment of MK-1775 and talazoparib, suggesting that ATP depletion was induced by the co-treatment. However, the sensitivity was different between the cell lines (Fig. 5D, Supplemental Fig. 2D).

## Discussion

In the MDS and AML cell lines, we tested the antitumor effects of targeting the DDR with a WEE1 kinase inhibitor (MK-1775) and a PARP inhibitor (talazoparib). In non-malignant eukaryotic somatic cells, WEE1 acts as a tumor suppressor [9]. However, WEE1 acts like an oncogene rather than a tumor suppressor in tumor cells. A genome-wide CRISPR screen of 563 cancer cell lines revealed that WEE1 was crucial for the viability of nearly all the cancer cell lines and that WEE1 was frequently overexpressed in both solid and hematologic malignancies [9]. Our study showed that the *WEE1* gene is highly expressed in AML patients compared with normal cells. Therefore, targeting WEE1 may be a candidate for AML treatments.

WEE1 inhibition has received a lot of attention in the last decade as a promising treatment for cancers, including hematologic malignancies, since it regulates the cell cycle [7]. The WEE1 kinase may act as a master regulator of the G2/M checkpoint, and WEE1 inhibitors can be used to prevent the activation of the G2/M cell cycle checkpoint [6]. WEE1 inhibition with 100 nM MK-1775 caused G1 arrest in SKM-1 cells, according to our findings (Fig. 4D). Since p53 is a key regulator of the G1 checkpoint, tumors lacking p53 rely solely on the G2 checkpoint after DNA damage [26]. Since SKM-1 is a *TP53*-mutated MDS cell line [27], the G1 cell cycle checkpoint may have been prevented by WEE1 inhibition in this study.

PARP-1 is a well-studied protein responsible for the majority of poly ADP-ribosylation reactions that use nicotinamide adenine dinucleotide as a substrate, thereby regulating various cellular functions [28]. Recent studies have found that PARP-1 localizes in mitochondria and that PARP-1 may play a role in maintaining mitochondrial homeostasis [28]. We found that MK-1775 and talazoparib inhibited the cell growth of the MDS and AML cells. According to our findings, the MK-1775 and talazoparib treatment increased caspase 3/7 activity while decreasing MMP activity (Fig. 5B C). The immunoblot analysis confirmed the activation of γ-H2AX, which shows an early cell response to the induction of DNA double-strand breaks (Fig. 4C). The MMP is an important indicator of mitochondrial activity since it reflects electron transport and is the driving force behind ATP production [24]. We also demonstrated that the ATP levels were reduced by the co-treatment of MK-1775 and talazoparib (Fig. 4B). Thus, the combination treatment attenuated the mitochondrial function and mediated cellular death in the MDS and AML cell lines.

WEE1 inhibitors were found to enhance the activity of chemotherapy or radiotherapy in tumors in preclinical and clinical studies [29, 30]. In preclinical studies, MK-1775 was found to have synergistic antitumor effects when combined with the PARP inhibitor olaparib and/or an ATR inhibitor [17]. An ongoing trial is testing the combination of MK-1775 with olaparib (ClinicalTrials.gov: NCT03579316) in solid tumors such as recurrent ovarian cancers. A previous report also demonstrated that the DNA damage repair interference by WEE1 inhibition combined with a low dose of cytarabine overcame the combined azacitidine and venetoclax resistance in AML [31].

## Conclusions

Although several new drugs have been introduced for patients with AML, patients with a refractory disease have no options, particularly after an HMA-based therapy [32]. Our study shows that the combination of a WEE1 inhibitor and PARP inhibitor had antitumor activities in MDS and AML cells. The combination of DNA damage and cell-cycle checkpoint inhibition improves therapeutic efficacy and is being proposed as a new option for high-risk MDS and AML patients.

## Electronic supplementary material

Below is the link to the electronic supplementary material.

**Supplemental Fig. 1**. Scatter plot of the cell cycle analysis stained by the propidium iodide stain from Fig. 4D

**Supplemental Fig. 2**. Effects of MK-1775 and talazoparib on MDS and AML cell proliferation. (A, B, C) MDS, AML, and non-cancer cells were incubated with MK-1775 or talazoparib for 48 or 72 h. Cell growth (A), caspase 3/7 activity (B), and cytotoxicity (C) were evaluated. (D) MDS and AML cell lines were incubated with MK-1775 or talazoparib for 72 h. The MMP was analyzed by a Mitochondria Staining Kit. Significance was expressed as *p < 0.05, **p < 0.01, ***p < 0.001, ****p < 0.0001, and ns, not significant

**Supplemental Fig. 3.** Uncropped immunoblot images from Figs. 2D and 3D

**Supplemental Fig. 4.** Uncropped immunoblot images from Fig. 4C

## Data Availability

The data in this manuscript are available upon request to the corresponding author.

## List of Abbreviations

MDS: Myelodysplastic syndrome
AML: Acute myeloid leukemia
IPSS: International prognostic scoring system
PARP-1: Poly (ADP-ribose) polymerase-1
ROS: Reactive oxygen species
HMA: Hypomethylating agents
AZA: Azacytidine
DDR: DNA damage response
LDH: Lactate dehydrogenase
RPMI 1640: Roswell Park Memorial Institute 1640
FBS: Fetal bovine serum
CO_2_: Carbon dioxide
shRNA: Short hairpin RNA
GEO: Gene Expression Omnibus
FAB: French American British
RA: Refractory anemia
RAEB: Refractory anemia with excess blasts
PBS: Phosphate-buffered saline
ATP: Adenosine triphosphate
CI: Combination index
MMP: Mitochondria membrane potential
NAD+: Nicotinamide adenine dinucleotide
